# Assessment of the potential risks in SD rats gavaged with genetically modified yeast containing the *cp4-epsps* gene

**DOI:** 10.3389/fvets.2024.1411520

**Published:** 2024-08-07

**Authors:** Bo Bi, Xuewei Fu, Xuewen Jian, Yu Zhang, Yizhi Jiang, Wuyi Zhou, Hui Zhao

**Affiliations:** ^1^Key Laboratory for Biobased Materials and Energy of Ministry of Education, Research Center of Biomass 3D Printing Materials, College of Materials and Energy, South China Agricultural University, Guangzhou, China; ^2^College of Food Science, South China Agricultural University, Guangzhou, China; ^3^Guangzhou Zhixin High School, Guangzhou, China

**Keywords:** CP4-EPSPS protein, biomarkers, metabolomics, gut microbiome, food safety

## Abstract

**Introduction:**

Despite the absence of definitive evidence indicating that the cp4-epsps gene and its resultant recombinant proteins have significant harmful effects on either human or animal health, the safety assessment of genetically modified (GM) crops expressing the CP4-EPSPS proteins has been controversial. This study endeavor was aimed at evaluating the potential risks posed by the CP4-EPSPS protein in transgenic crops, thereby contributing to the advancement of risk assessment methodologies in the context of genetically engineered crops.

**Methods:**

To ascertain the appropriate daily dosages for oral gavage administration, the expression levels of the CP4-EPSPS protein in a recombinant yeast were quantified. Subsequently, physiological and biochemical analysis, metabolomics, and metagenomic analysis were conducted based on a 90-day Sprague-Dawley (SD) rats feeding experiment, respectively, thereby enhancing the depth and precision of our risk assessment framework.

**Results:**

The results from the physiological and biochemical analysis, organ pathological, blood metabolism, gut microbiota, and correlation analysis of metabolites and gut microbiota revealed several biomarkers for further risk assessment. These biomarkers include clinical biochemical indexes such as total bilirubin (TBIL), direct bilirubin (DBIL), creatine kinase (CK), and lactate dehydrogenase (LDH); metabolites like Methionine, 2-Oxovaleric acid, and LysoPC (16:0); and gut microbiota including *Blautia wexlerae*, *Holdemanella biformis*, *Dorea* sp. *CAG 317*, *Coriobacteriaceae* and *Erysipelotrichaceae*.

**Conclusion:**

In conclusion, the risk can be significantly reduced by directly consuming inactivated recombinant CP4-EPSPS. Therefore, in everyday life, the risk associated with consuming GM foods containing recombinant CP4-EPSPS is substantially reduced after heat treatment.

## 1 Introduction

According to the global status of commercialized biotech/genetically modified (GM) crops in 2023, a total of 206.3 million hectares of biotech/GM crops have been grown commercially in 27 countries (1). Among the top 10 approved GM crops based on the International Service for the Acquisition of Agri-biotech Applications (ISAAA) GM approval database, four of them are herbicide tolerant (HT) crops expressing CP4-EPSPS, these include HT maize NK603, HT soybeans GTS 40-3-2, HT soybeans GA21 and stacked insect resistant and herbicide tolerant (IR/HT) maize MON88017 (2). The enzyme 5-enolpyruvylshikimate-3-phosphate synthase (EPSPS) plays a crucial role in the shikimate pathway in plants (3). Glyphosate and phosphoenolpyruvate (PEP) competitively interact with the EPSPS enzyme, leading to the inhibition of the production of three essential amino acids (phenylalanine, tyrosine, and tryptophan) and ultimately causing the death of the plants (4). Overexpressing the EPSPS protein in plants is one of the most effective breeding strategies for developing HT crops. The safety assessment of GM events primarily follows the principle of substantial equivalence from the OECD (Organization of Economic Co-operation and Development) and the 90-day rodent feeding experiment from EFSA (European Food Safety Authority) (5). Although there is no clear evidence of significant adverse impacts on human or animal health from the *cp4-epsps* gene and its recombinant protein (6, 7), the safety assessment of the GM crops expressing CP4-EPSPS is closely monitored, and unintended effects are one of the major concerns.

Metabolomics studies (8–10) have been widely used in comparing molecular characteristics and components of GM crops with their conventional comparators, helping to establish the substantial equivalence principle and identify potential unintended changes resulting from foreign gene insertion (11). Metabolomics is also a powerful tool for monitoring disease status and understanding disease pathogenesis (12). However, there have been few studies using metabolomics to evaluate the unintended effects on animals fed genetically modified crops.

The intestine also plays a crucial role in nutrition digestion and absorption (13), and intestinal health is essential for overall well-being. The dietary component, including GM ingredients such as CP4-EPSPS protein, can influence gut microbes. Therefore, it is important to investigate whether these GM ingredients can potentially affect the gut microbiota of animals and cause unintended effects. This study aims to assess the potential risks of the transgenic protein CP4-EPSPS and to demonstrate the feasibility of the assessment methods. We first constructed a recombinant yeast expressing CP4-EPSPS, broke it down to release the protein, and fed it to Sprague–Dawley (SD) rats. Based on comprehensive results from serum biochemistry, pathological sections, metabolomics, and gut microbiota, we thoroughly analyzed the potential impact of the CP4-EPSPS transgenic protein on the animal body. We particularly compared the assessment results of crude protein and denatured protein, finding that the potential risk significantly decreases after food is cooked, clarifying the potential connection between food processing methods and the biosafety of genetically modified crops. Together, our results emphasize the key role of the processing methods of genetically modified crops in risk control, providing a new perspective for food safety assessment.

## 2 Materials and methods

### 2.1 Strains and materials

The details about materials, reagents, and experimental animals are described in the Supplementary material.

### 2.2 Methods

#### 2.2.1 Identification of recombinant yeasts expressing CP4-EPSPS

To construct the recombinant yeast expression CP4-EPSPS, the *cp4-epsps* gene was optimized and synthesized according to Genebank KP212901.1, taking into account the codon bias of yeast, GC content, and the Kozak sequence of pPICZb vector. The *cp4-epsps* gene was inserted into the pPICZb vector at the SfuI and XbaI restriction sites. The recombinant yeasts are called pPICZb-epsps (pEB).

#### 2.2.2 Optimization of the recombinant yeast expression

We optimized the expression of the recombinant yeast, according to the optimized expression protocol, and the fermentation was scaled up to produce a larger quantity for subsequent analysis and oral administration in SD rats. The details are described in the Supplementary method 1.

#### 2.2.3 Parallel reaction monitoring analysis for CP4-EPSPS protein expression level in *Pichia* strains

To compare the expression level of recombinant CP4-EPSPS protein in GS115 with GM commodities, and provide a reference for setting the dose of recombinant yeast by gavage in rats. The expression level of CP4-EPSPS protein was analyzed and quantified by LC-PRMMS at Shanghai Applied Protein Technology Co. (14). All samples were detected by PRM and the raw data were analyzed using Skyline. Based on the quantitative information of the isotope-labeled peptides and the theoretical molecular weight of the target proteins, the target proteins were analyzed for absolute quantification of CP4-EPSPS protein per gram of sample.

#### 2.2.4 Physiological and biochemical analysis by gavage in SD rats

All SD rats were housed in a facility with controlled humidity (40–70%) and temperature-controlled (23 ± 2)°C on 12:12 h light: dark cycles under specific pathogen-free conditions. They had free access to food and water throughout the experiment. The animal procedures were approved by the Ethical Committee on Animal Care and Use of South China Agricultural University, China (Protocol Code: 2020B045). The experimental rats were randomly assigned into 3 groups, with 15 male SD rats in each group. We gavaged rats at a dose of 2.4 g/100 g/day (wet yeast/bw/day). The yeast was solid, and we diluted it into a 2.5–4 mL solution with sterile water and then gave it to the rats 7 days a week for 13 weeks (3 months). Each group received different treatment: crushed recombinant yeast with pPICZb as negative control (Control A), crushed recombinant yeast with pPICZb-epsps as the first experimental group (Group B), and crushed and boiled recombinant yeast with pPICZb-epsps (inactivated recombinant CP4-EPSPS protein) as the second experimental group (Group C).

Before dissection, each rat was fasted for 24 h and weighed. On experimental day 91, stool samples were collected using the sterile EP tube. The rats were then anesthetized with 5% chloral hydrate solution in water and dissected. Two blood samples were collected from the arteria cruralis, both of which were 2 mL. One for plasma using a sodium-heparinized tube to prevent coagulation was immediately snap-frozen at −80°C for metabolomics analysis. Another serum using an untreated tube was kept at room temperature for 1 h and then centrifuged for 15 min at 3000 r/min at 4°C, the resulting serum was ready for biochemical analysis. Additionally, organs such as the liver, spleen, kidney, brain, and heart were weighed and fixed in 10% neutral buffered formalin for histological analysis.

#### 2.2.5 Untargeted metabolomics detection

First, 400 μL of cold extraction solvent methanol/acetonitrile/H_2_O (2:2:1, v/v/v) was added to 100 μL sample to extract metabolites from plasma samples for LC–MS analysis. The quadrupole time-of-flight mass spectrometer (SCIEX Triple TOF 5600) was coupled to hydrophilic interaction chromatography via electrospray ionization to analyze polar metabolites for untargeted metabolomics (Shanghai Applied Protein Technology Co., Ltd. LC) (15). The Variable Importance for the Projection (VIP) value of each variable in the OPLS-DA model was calculated to indicate its contribution to the classification. Significance was determined using an unpaired Student’s *t*-test. VIP value>1 and *p* < 0.05 were considered statistically significant. The details about chromatography and mass spectrometry conditions and data analysis are described in Supplementary method 2–4.

#### 2.2.6 Metagenomic analysis of microbial communities

Total DNA was extracted from stool samples (*n* = 5, per group) using the Stool DNA Kit (TIANGEN, China). After sonication, the DNA sample was processed through various steps including end-polishing, A-tailing, ligation with adaptors, purification, and PCR amplification to construct the library. Once the library passed quality control, different libraries were pooled based on the desired concentration and target data volume of Illumina PE150 sequencing. The raw data underwent quality control and host filtering to obtain clean date^1^ (16). From the clean data, subsequent analyses were performed (17), including metagenome assembly, gene prediction, species annotation, abundance clustering analysis, PCA and NMDA dimensionality reduction analysis, Anosim analysis, and sample clustering analysis, to explore the differences in species composition and functional composition between samples (18).

#### 2.2.7 Correlation analysis

The relative abundances of four bacterial groups and the expression levels of 34 metabolites were analyzed using metagenomic sequencing and metabolomics analysis, respectively. A correlation matrix heatmap was generated by calculating the Spearman correlation coefficient between these two datasets in all experimental samples.

## 3 Results

### 3.1 Optimization of *cp4-epsps* gene expression parameters for recombinant yeast on a large scale

Various foreign protein expressions in *Pichia* system have been challenging due to various factors such as codon bias, media composition, inducer concentration, induction time, temperature, and so forth (19–21). According to our previous study, we found that induction time significantly impacts the production efficiency on a large scale. Thus, optimizing induction time is essential for the successful larger-scale shake-flask fermentation of *cp4-epsps* gene in *Pichia*.

**Figure 1:**
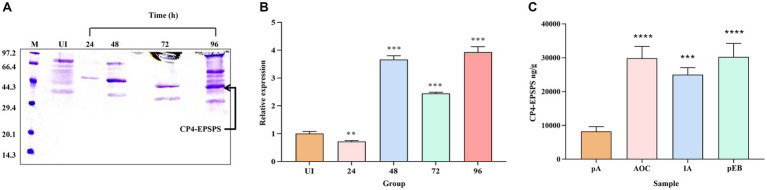
Comparison and assessment of CP4-EPSPS expression at different time points and quantitative analysis. **(A)** The cultures were induced by 0.5% methanol at 4 time points, respectively, (24 h,48 h,72 h, and 96 h) to compare the difference in the expression level. Protein samples were resolved in 12% SDS-PAGE and the amount of protein loaded in each well was 30 μL /well. *M* protein marker (kDa); *UI* uninduced. **(B)** Quantitative determination of protein in different periods (24 h, 48 h, 72 h, and 96 h). **(C)** LC-PRM/MS quantitative analysis. pA(UI) is the negative control before induction; MON89788 (AOC) and IA are maize commodities, pEB is recombinant yeast at the 48  h time period.

After 0.5% methanol induction and purification, an intense band at 47 kDa CP4-EPSPS protein could be observed at all induction time points (Figure 1A), and the assessment of the difference in expression of the protein at different induction time points confirms the maximum expression of CP4-EPSPS between 48 h and 96 h (Figure 1B). However, the purity of the target protein at 96 h is lower than at 48 h and 72 h which indicates the endogenous protein of the former probably was more abundant. In consideration of productive efficiency and expression level, the induction time at 48 h was selected as the optimal induction time point compared to 72 h.

### 3.2 The determination of *cp4-epsps* gene expression levels in samples

In this study, two endogenous peptides (LAGGEDVADLR and GVTVPEDR) of CP4-EPSPS protein in 10 different samples were analyzed using LC-PRM/MS quantitative analysis, the result showed that the target protein had a strong quantitative signal in 10 samples. Based on the quantitative information of the isotype-labeled peptides and the theoretical molecular weight of CP4-EPSPS protein, the absolute quantification for target peptides and target protein was carried out to determine the content of each sample (per gram) (Figure 1C) which would provide a reference for determining the dosing volume to be administered orally in rats.

The result of PRM showed CP4-EPSPS expression level of recombinant yeast (recombinant yeast (pEB)) after induction was equal to the sample of MON89788(AOC)and unnamed maize commodity (IA, a farm of Iowa state in the USA).

### 3.3 Physiological and biochemical analysis in SD rats

#### 3.3.1 Body weight and organ weights

**Figure 2:**
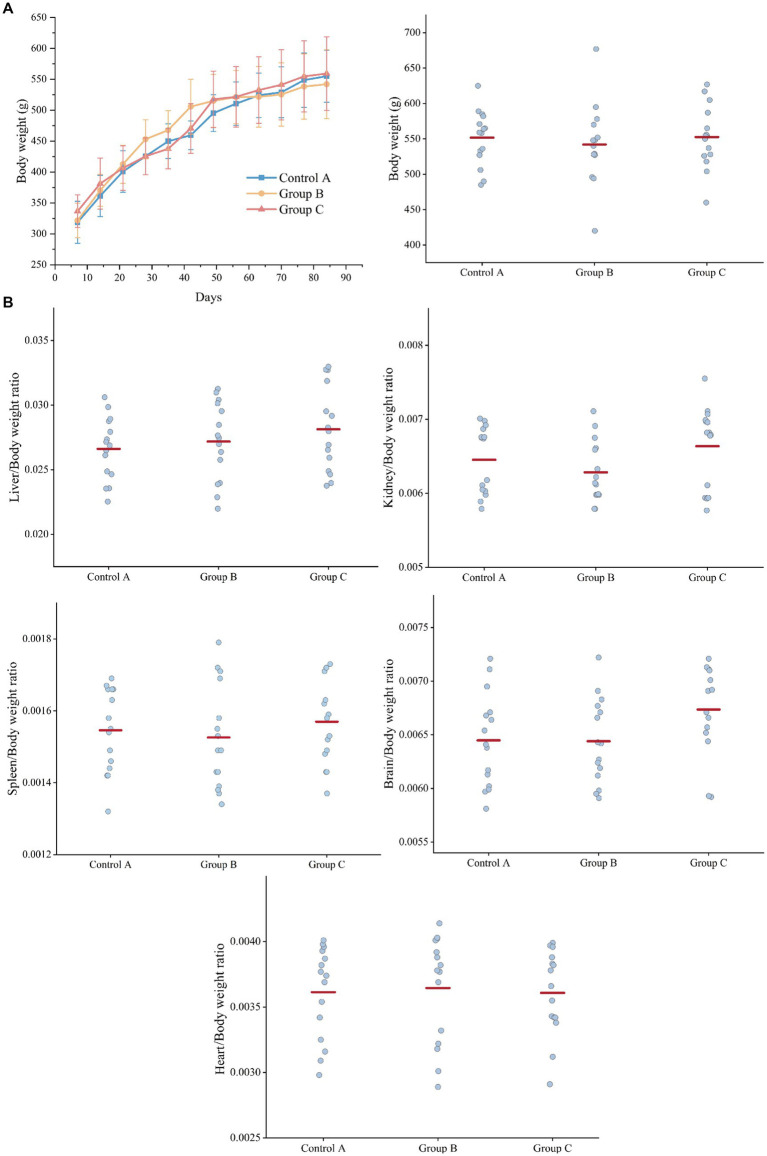
Weight comparison of three groups of rats. **(A)** Body weight. **(B)** Organ weight/Body weight ratio (liver, kidney, spleen, brain, heart). Control A: Control group of recombinant yeast crushed by pPICZb consumption; Group B: Experimental group consuming pPICZb-epsps crushed recombinant yeast; Group C: Experimental group consuming pPICZb-epsps (high temperature inactivated).

During the 90-day gavage toxicity study, 45 rats were dosed at 2.4 g/100 g (wet yeast/day) and observed for signs of illness once daily, all rats of the three groups looked quite healthy and behaved normally (Diet composition and feed consumption are shown in Supplementary Table S1). Compared to controls, the rats of three groups did not show a significant difference in body weight (bw) at different time points (7 d, 14 d, 21 d, 28 d, 35 d, 42 d, 49 d, 56 d, 63 d, 70 d, 77 d, 84 d, and 91 d) (Figure 2A).

At the end of the study, each rat was killed and a complete gross examination was performed. The Liver, heart, kidney, spleen, and brain were weighed, and the correlation of internal organ weight with body weight (Figure 2B). Overall, there was no statistically significant difference in organ weight between groups. This result suggested both the active and inactive CP4-EPSPS protein have had no obvious effect on the weight of rats.

#### 3.3.2 Histopathological analysis

**Figure 3:**
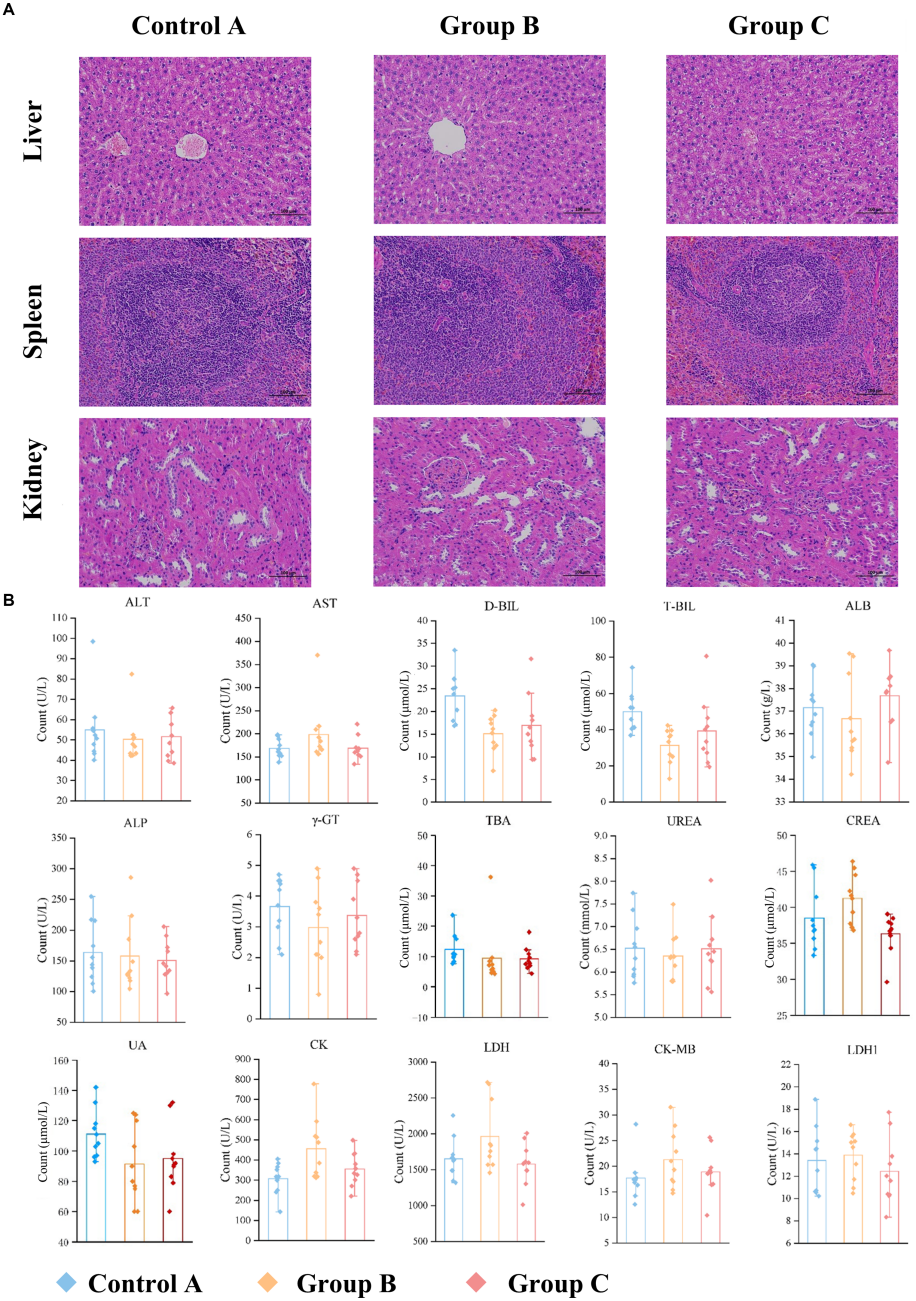
Comparison of organ and serum indexes in three groups. **(A)** Representative results of H&E staining images of organ sections (liver, spleen, kidney; original magnification: 200×). There is no visible difference. **(B)** Serum biochemical results.

Histopathological images of the organs such as the liver, spleen, and kidney (*n* > 3) are analyzed at 200× magnification to study the organ lesions. The results showed there was no lesion found on all images which suggested there is no visible difference between groups (Figure 3A).

#### 3.3.3 Analysis of blood biochemistry

Blood biochemistry of the blood gives us an indication of what is happening within the body. A serum sample was obtained from each rat on day 91. We analyzed 15 clinical measurements of Control A, Group B and Group C, including alanine aminotransferase (ALT), aspartate aminotransferase (AST), direct bilirubin (D-BIL), total bilirubin (T-BIL), albumin (ALB), alkaline phosphatase (ALP), glutamyl transferase (γ-GT), total bile acid (TBA), urea (UREA), creatinine (CREA), uric acid (UA), creatine kinase (CK), lactate dehydrogenase (LDH), creatine kinase isoenzyme (CK-MB) and lactate dehydrogenase isoenzyme (LDH1). The characteristics of the three groups are presented in Supplementary Table S2 and Figure 3B.

D-BIL and T-BIL were reduced significantly (*p* < 0.001, *p* < 0.001) in Group B (16.1, IQR: 11.9–20.3 μmol/L; 33.4, IQR:22.1–42.4 μmol/L) compared with Control A (23.4, IQR: 16.8–25.3 μmol/L;49.7, IQR:40.4–74.3 μmol/L), CK was increased (*p* < 0.05) in Group B (456.2, IQR: 312–777.8 U/L) compared with the A (307.9, IQR:143.2–405.2 U/L). Besides, CREA was increased significantly (*p* < 0.01) in Group B (41.2, IQR: 36.8–46.4 μmol/L) compared with C (36.3, IQR: 29.6–39.0 μmol/L), LDH in Group B (1962.6, IQR: 1455.7–2717.1 IU/L) was higher than Group C (1580.5, IQR: 1012.2–1941.9 IU/L) with *p* < 0.05.

### 3.4 Untargeted metabolomics analysis

#### 3.4.1 Differential analysis of different groups based on untargeted metabolomics

**Figure 4:**
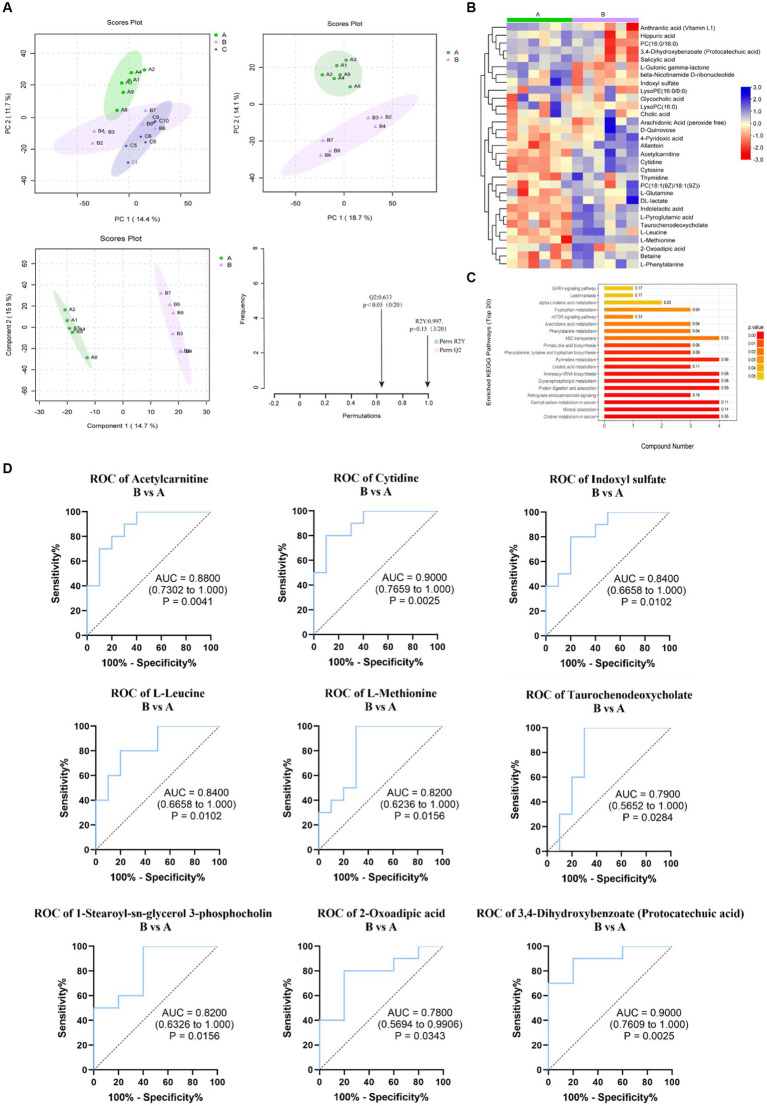
Metabonomic data analysis (Group B and Control A). **(A)** PCA plots and OPLS-DA scores and substitution tests. **(B)** Heat map of significantly altered metabolites in positive and negative detection mode. **(C)** KEGG pathway enrichment of differential metabolites. **(D)** Evaluation of the effectiveness of 30 metabolite indicators for classification and diagnosis. (The graph shows the 9 metabolites with the most significant indicators).

OPLS-DA was used to further analyze the similarities and differences in the samples after removing QC samples. In the both HILIC negative and positive ion detection modes, the rats’ blood metabolic patterns changed with different diets, as indicated by the trend of group clustering and separation in pairwise group comparisons (Figure 4A). However, there was no significant difference between Group B and C, as shown in Supplementary Figure S1.

According to the OPLS-DA, the VIP (variable importance in projection) obtained was used to discover the different metabolites with biological significance. To identify significantly different metabolites better that may play an important role under different feeding, pairwise comparisons between groups were performed. When Group B was compared with Control A, 30 significantly different metabolites (VIP > 1.0, *p* < 0.05) (Supplementary Table S3) and 30 different metabolites (VIP > 1.0, 0.05 < *p* < 0.1) (Supplementary Table S4) were altered. Among these metabolites, 26 total different metabolites could have relationships with the KEGG metabolic pathways related to human diseases (choline metabolism in cancer, central carbon metabolism in cancer), digestive system (mineral absorption, protein digestion, and absorption), amino acid metabolism (phenylalanine/tyrosine/tryptophan biosynthesis, valine/leucine/isoleucine biosynthesis and degradation and Tryptophan metabolism) and lipid metabolism (Primary bile acid biosynthesis, Linoleic acid metabolism, Arachidonic acid metabolism, and alpha-linolenic acid metabolism), environmental information processing-membrane transport (ABC transporters) and so on. The top 20 enriched pathways in B compared with A are shown in Figure 4C. Among 26 metabolites related to metabolism, 17 were upregulated while 9 were downregulated in Group B than in Control A (Figure 4B). When Group C was compared with Group B, 25 significantly different metabolites (VIP > 1.0, *p* < 0.05) and 11 differential metabolites (VIP > 1.0, 0.05 < *p* < 0.1) were provided in the Supplementary Tables S5, S6. 17 metabolites are related to the metabolism pathways such as human diseases, amino acid metabolism, digestive system, carbohydrate metabolism and so on. Among them, 5 metabolites were upregulated while 12 were downregulated. Then, we used stepwise logistic regression models to classify those metabolites of paired groups (Figure 4D; Supplementary Figure S2).

### 3.5 Gut microbiota

Because of oral administration, whether recombinant protein CP4-EPSPS will affect gut microbiota associated with metabolites differentiation. We further investigated the gut microbiota using fecal 16SrDNA.

**Figure 5:**
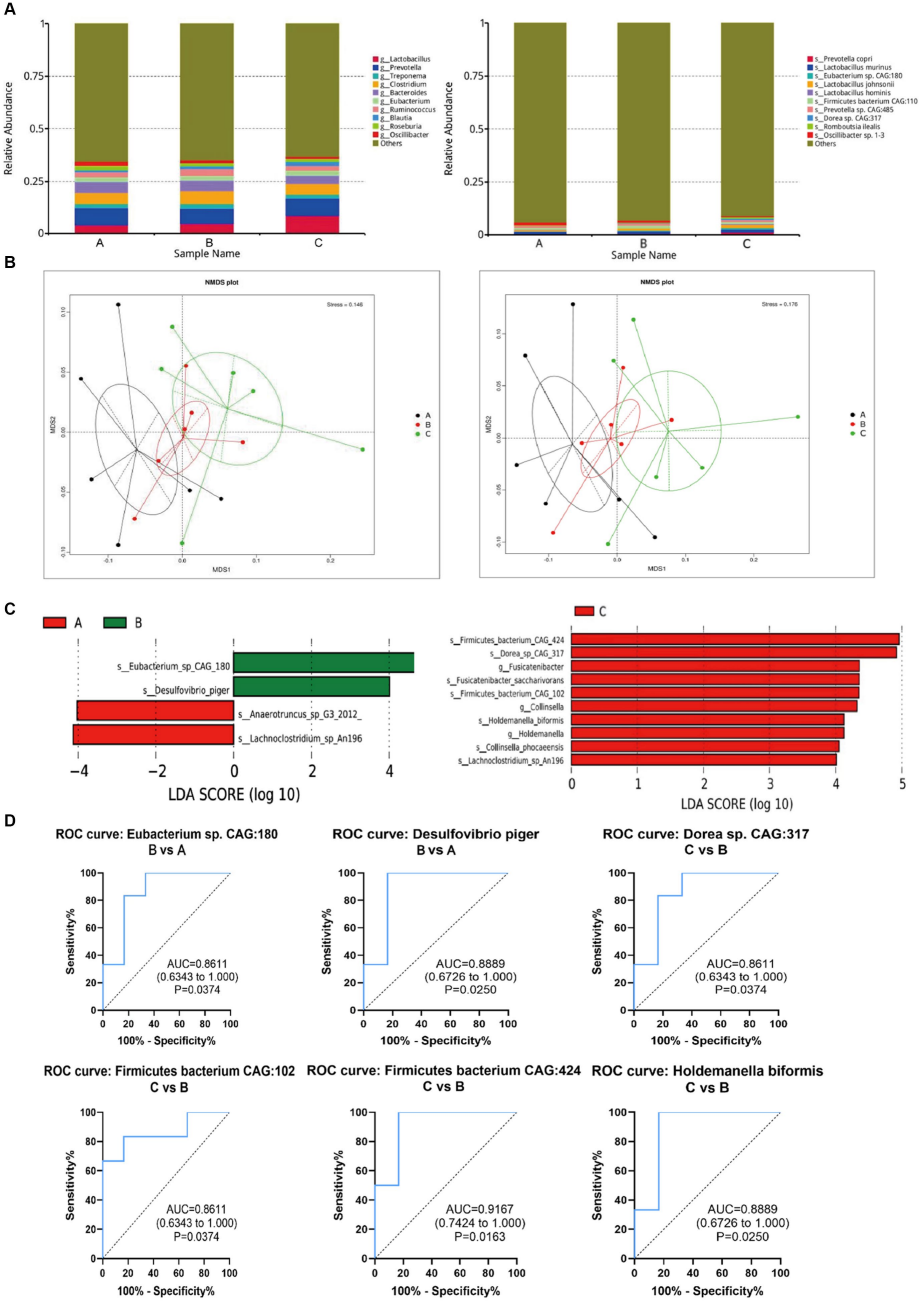
Changes of intestinal microorganisms (three groups of rats). **(A)** Relative abundance analysis of the top 10 gut microbiota at the genus and species level. **(B)** Dimension-reducing analysis based on species abundance. **(C)** Column chart of LEfSe analysis of gut microbes of the pairwise comparisons between groups. **(D)** Evaluation of the effectiveness of gut microbiota biomarkers of pairwise comparison.

According to the annotation results at genus levels, we plotted the histograms for each group. At the genus levels, the results showed that *Lactobacillus*, *Prevotella*, *Treponema*, *Clostridium*, *Bacteroides*, *Eubacterium*, *Ruminococcus*, *Blautia*, *Roseburia*, *Oscillibacter* were the top 10 microbiomes with maximum relative abundance. The top 10 microbiomes with maximum relative abundance at the species level are shown in Figure 5A.

Then, the dimension-reducing analysis based on species abundance was performed. Although each sample is not a high-similarity item within a group, the Non-Metric Multi-Dimensional Scaling (NMDS) (Figure 5B) presented that the gut microbiota of three groups had significantly diverged from each other at genus and species levels (stress points = 0.146 and 0.176, respectively).

To screen the significantly different species as biomarkers between groups. The LDA (linear discriminant analysis) was performed to achieve dimensionality reduction and evaluated the effect of differential species to obtain the LDA score of the pairwise comparisons between groups. As shown in Figure 5C, the species as biomarkers that are statistically different between Control A and Group B included *Eubacterium*, *Desulfovibrio*, *Anaerotruncus*, and *Lachnoclostridium*. The differential species between Group C and B included *Firmicutes* bacterium, *Dorea*, *Fusicatenibacter*, *Fusicatenibacgter saccharivorans*, *collinsella*, *Holdemanella biformis*, *Collinsella phocaeensis*, and *Lachnoclostridium*.

Based on ROC analysis comparing Group B to Control A, *Eubacterium* sp. CAG180 (*p* = 0.03) and *Desulfovibrio piger* (*p* = 0.02) can be considered as potential biomarkers for studying the impact of CP4-EPSPS on the gut microbiota of SD rats. Additionally, when comparing Group C to Group B, *Dorea* sp. CAG 317 (*p* = 0.03), *Firmicutes* bacterium CAG102 (*p* = 0.02), *Firmicutes* bacterium CAG424 (*p* = 0.02), and *Holdemanella biformis* (*p* = 0.02) can serve as indicative markers for this purpose as well. Meanwhile, in comparison with Control A, the relative abundance of these microorganisms such as *Dorea* sp. CAG317, *Erysipelotrichales*, *Blautia wexlerae*, *Holdemanella biformis* in Group B significantly increased (*p* < 0.05) which probably provides valuable insights into the effects of CP4-EPSPS on gut microbiota composition (Figure 5D).

### 3.6 The correlation analysis of metabolites and gut microbiota

**Figure 6:**
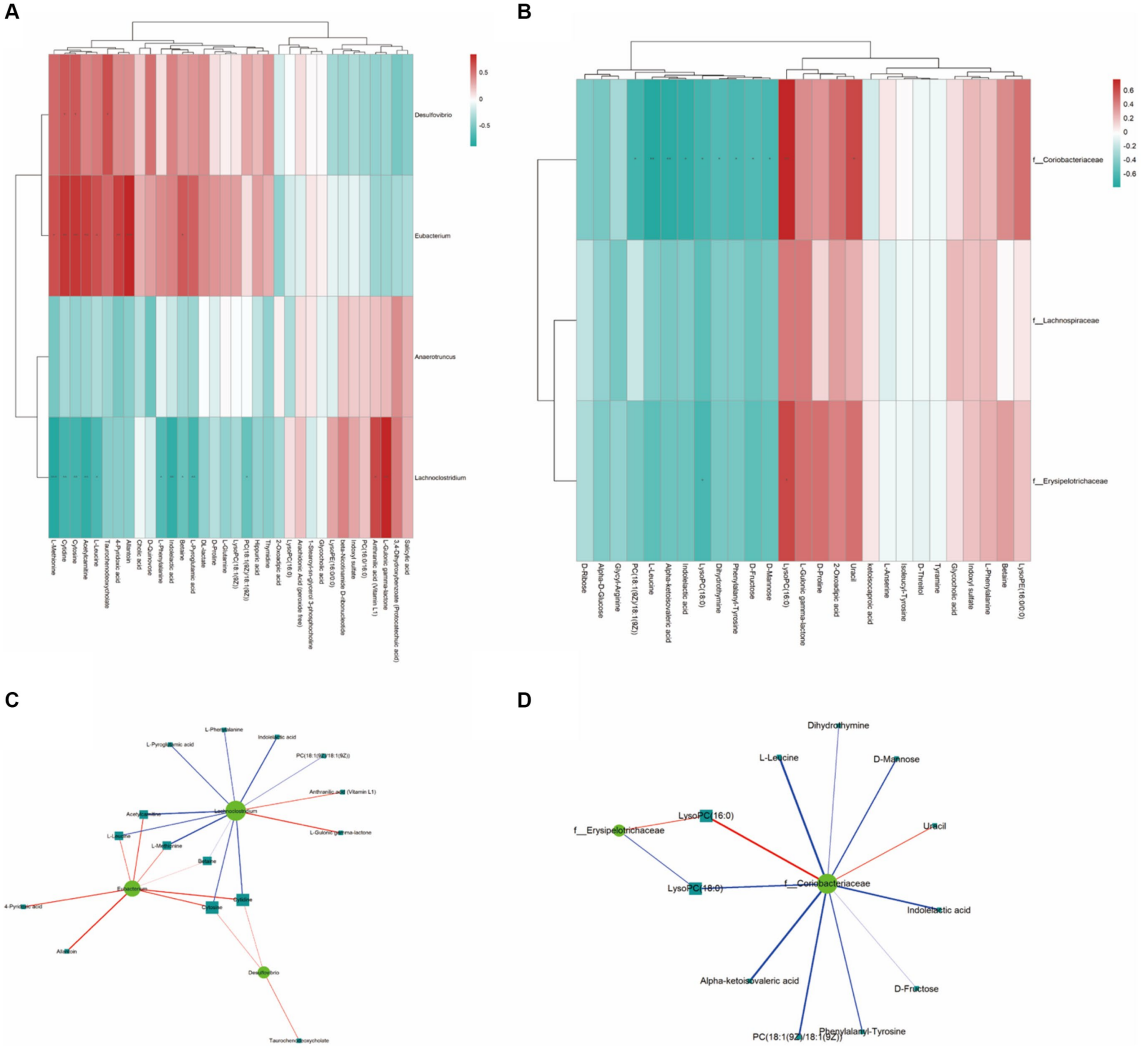
The Spearman correlation analysis. **(A,B)** are the Spearman correlation hierarchical clustering analysis to intuitively reflect the similarities and differences in expression patterns between significantly different bacterial communities and metabolites. Each row in the hierarchical clustering heatmap represents a significantly different bacterial genus, and each column represents a significantly different metabolite. Each cell in the hierarchical clustering heatmap contains two pieces of information (correlation coefficient *r* and *p* value). The correlation coefficient r is represented by color; red represents a positive correlation with *r* > 0, while blue represents a negative correlation with *r* < 0. The darker the color, the stronger the correlation. The *p* value reflects the level of significance of the correlation, with * indicating *p* value <0.05, ** indicating *p* value <0.01, and *** indicating *p* value <0.001. **(C,D)** are the Spearman correlation analysis network diagram for studying the correlation between significantly different bacterial communities and significantly different metabolites. The circles in the graph represent bacterial genera with significant differences, and the squares represent metabolites with significant differences. The color of the lines represents the positive or negative correlation coefficient between them (blue represents negative correlation, red represents positive correlation), and the thickness of the lines is proportional to the absolute value of the correlation coefficient. The size of the nodes is positively correlated with their degree, that is, the larger the degree, the larger the node size.

The analysis of the Spearman rank correlation test for 4 significantly differential bacteria genera with the maximum relative abundance (LEfSe LDA > 2 and *p* < 0.05) and 34 significantly differential metabolites (VIP > 1.0 and *p* < 0.05) between A and B was further performed including matrix Heat map analysis and heatmap analysis correlation network analysis (Figures 6A,C). The same method was used for the comparison of C and B, the results are shown in Figures 6B,D.

As a result, using the Spearman statistical analysis method, a total of 13 positive correlations and 10 negative correlations were found in the significant correlations between the bacterial groups and metabolites from Control A and Group B. As for Group C vs. B, a total of 3 positive correlations and 10 negative correlations were found.

According to the correlation between gut microbiota and metabolites, we found that the concentrations of metabolites including Acetylcarnitine, cytidine, L-leucine, L-Methionine, and Taurochenodeoxycholate are positively correlated with *Eubacterium* sp. CAG180 and *Desulfovibrio piger* (Figure 6A). Meanwhile, *Coriobacteriaceae* family and *Erysipelotrichaceae* family are negatively correlated with L-leucine, D-Mannose, Indoleacetic acid, D-Frustose, PC (18:1(9Z)/18:(9Z)), Alpa-ketoisovaleric acid, and LysoPC (18:0) (Figure 6B). Additionally, both microbial families mentioned above are positively correlated with LysoPC (16:0).

## 4 Discussion

### 4.1 The significance of the determination of *cp4-epsps* gene expression levels in recombinants

To maintain the genetic consistency of GM components, we engineered a recombinant yeast strain contains *cp4-epsps* gene. Simultaneously, to achieve a feeding dose comparable to that of GM crops, we quantified the expression level of *cp4-epsps* in the recombinant yeast and determined the appropriate feeding dose for SD rats. This ensures that the GM protein dose from recombinant yeast can reach levels similar to those used in conventional GM crop studies.

The results obtained using PRM demonstrate that under appropriate expression conditions, the expression level of *cp4-epsps* gene in the recombinant yeast is equivalent to that found in GM soybean commodities (AOC) and IA (Figure 1C). Considering an adult SD rat weighs approximately 250 grams and consumes around 30–40 g/day, three different doses for GM components in their diet can be set at low (11% of the total diet), medium (22% of the total diet), and high doses (33% of the total diet) (22). In our study, we selected a medium dose level of approximately 20%, which corresponds to about 2.4 g/100 g/day (wet yeast/rat/day). Based on protein quantification results, we determined the feeding dose of recombinant yeast according to rat weight (Supplementary Table S7). Compared with traditional feeding methods, gavage feeding minimizes the waste of GM components and ensures full delivery into the rat’s gastrointestinal tract. Therefore, our methods and results are theoretically consistent with traditional approaches, suggesting the reliability of our evaluation.

### 4.2 The safety assessment based on physiological and biochemical parameters

Our results showed that Group B had significantly lower levels DBIL and TBIL compared to Control A (*p* < 0.01), while ALT and AST levels were within the normal range. Conversely, there were no significant differences in DBIL and TBIL values between Group C and Control A (23). Many studies have shown a potential association between abnormal changes in TBIL and DBIL levels and various diseases (24). For instance, a study on primary Sjogren’s syndrome (pSS) observed reduced levels of TBIL, DBIL, and IBIL in pSS patients compared to healthy controls, along with higher erythrocyte sedimentation rate (ESR), whereas no significant differences were found for ALT and AST. Given that ESR is an inflammatory factor negatively correlated with TBIL and DBIL, this result suggests that the decrease in TBIL and DBIL may play a crucial role in protecting against inflammation-related diseases. Similarly, a previous study on Systemic Lupus Erythematosus (SLE) reported significantly lower concentrations of TBIL and DBIL in SLE patients than in the healthy population, indicating their potential antioxidant and anti-inflammatory effects (25). Therefore, further research is needed to determine if consuming raw ground GM yeast can trigger an inflammatory response. However, our findings confirm that boiled ground GM yeast does not adversely affect rats, aligning with Control A results.

When comparing Group B with Control A, differences were also observed in CK and LDH. Among the 10 samples from Group B, 3 samples (30%) exhibited abnormalities in both CK (>480 U/L) and LDH (>2,400 U/L). CK and LDH are commonly used as markers of tissue damage. Generally, elevated serum CK levels can indicate tissue damage (26), and LDH catalyzes the conversion of pyruvate to lactate during periods of high muscular activity (27). The 30% of rats in Group B that showed abnormalities in CK and LDH could be indicative of tissue organ damage, although no abnormal tissue lesions were found in the pathological sections of the liver, spleen, kidney, and heart tissues. This indicates that CK and LDH are significant biochemical markers that require further study. All results from Group C were within the normal range, indicating that the deactivated recombinant protein CP4-EPSPS did not affect the rats’ health.

### 4.3 Metabolomics data analysis

Combining the ROC analysis of Group B versus A and KEGG pathway analysis, among the 9 potential biomarkers (Figure 4D), L-leucine and L-Methionine are associated with central carbon metabolism cancer and the digestive system (protein digestion and absorption, mineral absorption), 2-Oxoadipic acid and PCA are related to amino acid metabolism, and cytidine to pyrimidine metabolism.

In a pairwise comparison of Groups C, B, and A, the concentration of the potential biomarker 2-Oxoadipic acid followed a C > B > A pattern, while LysoPC (16:0) followed a B < C < A pattern. L-anserine, D-mannose, and D-fructose showed consistent concentration changes, with no significant difference between Group B and Control A, but a significant downregulation in Group C. The yeast cell lysates fed to rats differed in that Group C was boiled, denaturing the protein, while Group B and A were not. We speculate that changes in L-Anserine, D-mannose, and D-Fructose may be due to active yeast cell nutrients, not the recombinant CP4-EPSPS protein.

#### 4.3.1 The analysis of the potential impact of recombinant CP4-EPSPS on central carbon metabolism and choline metabolism in cancer

According to Supplementary Table S3, there were significant differences in the levels of four amino acids between Group B and Control A, including L-leucine (VIP = 3.15, FC = 1.19, *p* < 0.01), L-methionine (VIP = 1.71, FC = 1.61, *p* = 0.03), glutamine (VIP = 1.01, FC = 1.26, *p* = 0.045), and L-valine (VIP = 4.03, FC = 1.12, *p* = 0.03). Furthermore, based on ROC analysis, L-methionine and L-leucine were screened out and could serve as potential biomarkers for assessing the potential risk of recombinant CP4-EPSPS protein in rats. Methionine, in particular, showed a significant increase in concentration, with an upregulation of 1.61-fold in Group B compared to Control A. While the change in leucine concentration was less pronounced. This suggests a lower potential risk associated with elevated Leucine levels from consuming yeast recombinant CP4-EPSPS protein.

Decades of research have demonstrated the important roles of amino acids in cancer metabolism, providing a foundation for the growth of cancer cells. This often involves amino acids such as glutamine, glutamate, leucine, and methionine (28). Amino acids like glutamate and branched-chain amino acids (BCAAs) (Leucine, Valine, Isoleucine) can fuel TCA cycle intermediates, releasing ATP to provide the energy necessary for tumor formation. Amino acids can also influence reactive oxygen species (ROS) homeostasis and epigenetic regulation through methylation and acetylation, thereby enhancing tumor invasiveness. For instance, methionine provides a methyl group for methylation processes. Methionine adenosyltransferase catalyzes the formation of S-adenosylmethionine (SAM) from methionine and ATP to provide a methyl group for DNA methyltransferases (DNMTs) and histone methyltransferases (HMTs). Research on non-small cell lung cancer tumor-initiating cells (TICs) has shown that TICs exhibit high methionine cycling flux and significant dependence on exogenous methionine (29). The enhanced methionine cycling leads to SAM over-supply, which in turn causes high DNA methylation and inappropriate gene silencing, as well as abnormal histone methylation and enhanced tumor growth. Therefore, the observed changes in methionine concentration in Group B, based on this research, warrant further investigation.

Simultaneously, to utilize amino acids, specific transporters must be present in the cell membrane to ensure the absorption and movement of amino acids between tissues (30). Certain transporters are preferred by tumor cells to meet their needs, as evidenced by the overexpression of selected transporters associated with specific types of cancer (31). Therefore, it is also recommended to use proteomics in conjunction with other methods for further investigation of cancer risk.

According to the results from the pairwise comparisons among the three groups, the concentration changes of LysoPC (16:0), which is involved in glycerophospholipid metabolism, followed the pattern of B < C < A. This indicates that the concentrations of LysoPC (16:0) in Groups B and C were significantly lower than those in Control A. The concentrations of LysoPC (18:1(9Z)), LysoPE (16:0/0:0), and PC (16:0/16:0) also decreased. According to the ROC analysis between Groups C and B, LysoPC (16:0) has the potential to serve as a biomarker with significant differences for assessing the potential risk associated with recombinant CP4-EPSPS protein.

Previous studies have reported that, compared with healthy controls, lower concentrations of LysoPCs were detected in breast cancer patients. It has also been reported that the reduction in levels of LysoPC (16:0) and LysoPC (18:0) is associated with a decrease in PC levels in liver cancer tissues. These two LysoPCs are the most abundant forms found in plasma, and their decreased levels largely lead to the overall decrease in total LysoPC concentration in plasma among breast cancer patients. This may be linked to the inflammatory activation status of cancer patients (32).

During the screening of plasma biomarkers for myocardial infarction, researchers observed that due to the close correlation between acute myocardial infarction and glycerophospholipid and glycerolipid metabolic pathways (33), LysoPC (16:0) and LysoPC (18:0) demonstrated a downward trend. However, after surgical treatment, LysoPC (16:0) can be restored to levels similar to the control group.

Therefore, based on the relationship between LysoPC (16:0) and disease, the integration of lipid metabolism and cardiac tissue metabolite analysis can provide additional insights into the safety assessment of recombinant protein CP4-EPSPS consumption. Interestingly, the results of our study show that the decrease in the concentration of LysoPC (16:0) in Group C is smaller. It can be inferred that the denaturation of the recombinant protein induced by boiling can mitigate the significant reduction in LysoPC (16:0), thereby reducing potential risks.

#### 4.3.2 Analysis of the potential impact of recombinant CP4-EPSPS on amino acid metabolism

Quan et al. (34) discovered that the introduction of exogenous free Nε-(carboxymethyl) lysine (CML) significantly altered the blood concentrations of 25 metabolites, including 2-oxo adipic acid, tryptophan, and quinolinic acid, all of which are implicated in diabetes development and complications. In their study on Behcet’s disease, Cui et al. (35) identified a significant differential expression of 2-oxo adipic acid in the sweat metabolome but found no such differences in serum samples.

Our research findings indicate that compared to Control A, there was a marked upregulation of 2-oxo adipic acid (VIP = 12.198; FC = 1.44; *p* < 0.05) in Group B. Furthermore, when comparing Group C to Group B, there was also a significant upregulation of 2-oxo adipic acid (VIP = 6.26; FC = 1.57; *p* < 0.05). The relationship among these three groups follows the pattern C > B > A. This suggests that regardless of its activity status, the recombinant protein CP4-EPSPS has a substantial influence on altering the levels of 2-oxo adipic acid. Given that 2-oxo adipic acid is a crucial metabolite in the metabolism of tryptophan or lysine (36), the potential risk associated with increased concentration due to CP4-EPSPS warrants further attention. However, there is currently no definitive evidence to suggest that an increase in 2-oxo adipic acid alone is associated with disease progression when the concentrations of tryptophan and lysine are normal. Therefore, to assess potential risks related to elevated levels of this metabolite, it would be beneficial to combine it with an analysis of other metabolites associated with tryptophan.

### 4.4 Regulatory effect of recombinant CP4-EPSPS on gut microbiota

A study conducted by Liu (37) demonstrated that the health conditions of obese patients across various countries could be ameliorated by augmenting the abundance of six bacterial species, including *Eubacterium* sp. *CAG180*, and diminishing the abundance of four bacterial species, such as *Dorea lognicatena*. Consequently, *Eubacterium* sp. *CAG180* is considered a beneficial broad-spectrum taxonomic biomarker, while *Dorea lognicatena* is deemed a detrimental broad-spectrum taxonomic biomarker. *Desulfovibrio piger*, a prevalent sulfate-reducing bacterial strain in the human digestive tract, exhibits a multitude of functions. For instance, it can aid the body in nutrient absorption and inflammation suppression by modulating the immune system (38). It can also contribute to a reduction in muscle endurance and is linked to chronic inflammatory diseases and cancer (39). Hosomi et al. (36) discovered that the oral administration of *Blautia wexlerae* (*B. wexlerae*) can trigger metabolic alterations in mice, exert anti-inflammatory effects, and decrease the probability of obesity and diabetes induced by a high-fat diet.

In our research, when compared to Control A, there was an increase in the abundance of beneficial bacteria such as *Eubacterium* sp. *CAG180* in Group B, along with a simultaneous increase in the abundance of *Desulfovibrio piger*. Therefore, the risk associated with the recombinant CP4-EPSPS protein of *Eubacterium* sp. *CAG180* and *Desulfovibrio piger* must be evaluated in conjunction with the specific symptoms exhibited by SD rats.

D-psicose possesses distinctive physiological functions and potential health advantages, such as reducing fat accumulation, decreasing postprandial blood glucose levels, inhibiting hepatic lipase activity, eliminating ROS, treating atherosclerosis, enhancing insulin resistance, and providing neuroprotection. D-psicose is primarily synthesized biologically, with D-psicose 3-epimerase playing a pivotal role. *Dorea* sp. *CAG317* exhibits high D-tagatose 3-epimerase activity, facilitating the transformation from D-glucose to D-psicose (40). Upon comparing Group C with Group B, an upregulation of *Dorea* sp. *CAG317* was observed, suggesting a potential increase in intestinal D-psicose 3-epimerase activity. This could promote the conversion of D-glucose to D-psicose, thereby contributing to health benefits.

*Firmicutes* bacteria serve pivotal functions in the intestine, including the synthesis of vitamins, the promotion of health, and the resistance against harmful bacteria. Specifically, *Holdemanella biformis* (*H. biformis*) is capable of producing short-chain fatty acids (SCFAs), which regulate protein acetylation and tumor cell proliferation by inhibiting the activation of calcineurin/NFATc3 (41). Additionally, *H. biformis* is known to optimize the sensitivity of the GLP-1 (glucagon-like peptide 1) system in type 2 diabetic obese patients (19). It also can mitigate hyperglycemia, enhance oral glucose tolerance, and restore hepatic gluconeogenesis and insulin signaling in obese mice. Consequently, the increased abundance of *H. biformis* in Group C is advantageous for the health of the intestinal ecosystem.

Therefore, although the pairwise comparison results between the three groups are not sufficient to indicate that recombinant CP4-EPSPS presents a potential risk to the intestinal microbiota, it can be affirmed that the intake of denatured and inactivated recombinant CP4-EPSPS protein in Group C can significantly reduce this uncertainty. The inactivated recombinant protein only functions as a nitrogen and carbon source involved in metabolism, which is advantageous for enhancing the abundance of beneficial bacteria such as *B. wexlerae*, *H. biformis*, and *Dorea* sp. *CAG 317* in the gut, thereby promoting the health of the organism.

### 4.5 Correlation between metabolites and composition of gut microbiota

The correlation between gut microbiota and metabolism reveals that the elevated concentrations of metabolite markers such as Acetylcarnitine, Cytidine, L-leucine, L-Methionine, and Taurochenodeoxycholate in Group B could be linked to fluctuations in the abundance of *Eubacterium* sp. *CAG180* and *Desulfovibrio piger*. Notably, the fluctuations in Leucine and Acetylcarnitine concentrations are minor, while the changes in L-methionine, Cytidine, and Taurochenodeoxycholate concentrations are more pronounced. The elevated concentration of L-methionine is associated with a higher risk, suggesting that the consumption of recombinant CP4-EPSPS protein might influence rat metabolism by altering the abundance of different microorganisms in the rat gut. Consequently, in addition to focusing on the alterations in methionine concentration and its associated transporters, alterations in the core gut microbiota should be regarded as a potential risk related to the intake of recombinant CP4-EPSPS protein. Furthermore, probiotic intervention could potentially reduce the risk associated with the consumption of this protein.

The study of the relationship between gut microbiota and metabolism in Groups C and B shows that an increased abundance of the Coriobacteriaceae and Erysipelotrichaceae families in Group C resulted in a decrease in the biomarker metabolite L-Leucine and an increase in LysoPC (16:0) concentration, significantly reducing the risk for Group C. Evidence suggests that Coriobacteriaceae and Erysipelotrichaceae are markers of recovery after probiotic intervention (42), and the abundance of Coriobacteriaceae is closely associated with health improvement mechanisms facilitated by exercise (43, 44). In conclusion, the correlation analysis between metabolite biomarkers and gut microbiota markers suggests that the health status of rats in Group C is superior to that in Group B, which is consistent with the previous conclusion.

## 5 Conclusion

In this research, numerous biomarkers were pinpointed for enhanced risk evaluation. These biomarkers encompass clinical biochemical indices such as TBIL, DBIL, CK, and LDH; metabolites like Methionine, 2-Oxovaleric acid, and LysoPC (16:0); and gut microbiota including *Blautia wexlerae*, *Holdemanella biformis*, *Dorea* sp. *CAG 317*, *Coriobacteriaceae*, and *Erysipelotrichaceae*. The study proposes that a comprehensive assessment of potential risks associated with recombinant proteins can be achieved by integrating biomarkers, tryptophan metabolism, and proteomics. Concurrently, it was also validated that the risk can be significantly mitigated by directly consuming inactivated recombinant CP4-EPSPS. In routine life, GM foods usually undergo heat treatment, which considerably diminishes the risk associated the consuming GM crops containing recombinant CP4-EPSPS.

## Data availability statement

The original contributions presented in the study are publicly available. This data can be found here: NCBI BioProject, PRJNA1135459.

## Ethics statement

The animal study was approved by the Ethical Committee on Animal Care and Use of South China Agricultural University, China (Protocol Code: 2020B045). The studies were conducted in accordance with the local legislation and institutional requirements.

## Author contributions

BB: Conceptualization, Data curation, Methodology, Writing – original draft. XF: Formal analysis, Software, Writing – original draft. XJ: Investigation, Software. YZ: Investigation, Software. YJ: Formal analysis. WZ: Conceptualization, Methodology, Writing – review & editing. HZ: Conceptualization, Formal analysis, Funding acquisition, Methodology, Project administration, Supervision, Writing – review & editing.

## Footnotes

^1^https://github.com/OpenGene/fastp
