# Supplementary material for: Assessment of the potential risks in SD rats gavaged with genetically modified yeast containing the cp4-epsps gene
Source: Front Vet Sci. 2024 Aug 7;11:1411520. doi: 10.3389/fvets.2024.1411520 (PMC11335726; doi:10.3389/fvets.2024.1411520)
Supplement: Supplementary file 1 [file Data_Sheet_1.docx]

Supplementary Material

# Supplementary Materials and methods

## Supplementary Materials.

The pPICZb vector (Invitrogen, V190-20) was used to express CP4-EPSPS protein. Escherichia coli (E. coli) strains TOP10 were suitable for propagation of the pPICZb vectors, and transformants were selected on low salt LB agar plates containing 25 μg/mL Zeocin™. Pichia strains GS115 (Mut+) are suitable for expressing the pPICZb vectors, and GS115 transformants were purified on YPDS plates containing 50 μg/mL Zeocin™. The HT soybean MON89788 powder (AOC) was obtained from Shenzhen Zhuoyue Biotechnology Co., LTD, and the random maize commodity (IA) was from the Boon farm of Iowa State, USA. SD(SPF) rats weighing 80-100 g were purchased from the Laboratory Animal Center of Southern Medical University (Experimental animal production license number is SCXK(Guangdong)2021-0041).

## Supplementary Method 1. Optimization of the recombinant yeast expression

To optimize the expression and conduct larger-scale shake-flask fermentation of the recombinant yeasts, a single colony of pEB yeast was inoculated in 10 mL of YPD medium and incubated at 28°C for 24 h. The cells were then precipitated and resuspended in MMH medium to an OD_600_ of 1.0. Subsequently, anhydrous methanol was added every 24 h (24,48,72, and 96 h) to a final concentration of 0.5%. The yeast cells were collected, centrifuged at 3000 r/min for 5 min, weighed, mixed with NBB (Natural Binding Buffer), and sonicated under ice bath conditions to break them.

After fragmentation, the cell lysates were centrifuged at 3000 r/min for 10 min at 4°C. The supernatant was transferred to a ProBond nickel column (Invitrogen) for adsorption. Then, NEB buffer was used for elution. The final products from different induction times were detected SDS-PAGE electrophoresis.

## Supplementary Method 2. Chromatographic

LC separation was on HILIC and RPLC of an Agilent 1290 Infinity LC UHPLC.HILIC column using a gradient of solvent A (25 mM ammonium acetate and 25 mM ammonium hydroxide in water) and solvent B (acetonitrile). The gradient was 95% B for 1 min, linearly reduced to 65% in 13 min, then reduced to 40% in 2 min and kept for 2 min, and then increased to 95% in 0.1 min, with a 5 min re-equilibration period employed. HSS T3 column using a gradient of solvent A (0.1% formic acid for positive ion mode; 0.5 mM ammonium fluoride in water for negative ion mode) and solvent B (0.1 vol% formic acid-acetonitrile for positive; acetonitrile for negative). The gradient was 1% B for 1.5 min, linearly increased to 99% in 12.5 min and kept for 3.5 min, then reduced to 1% in 0.1 min and then kept for 3.5 min.

## Supplementary Method 3. Q-TOF Mass Spectrometry

For untargeted metabolomics of polar metabolites, extracts were analyzed using a quadrupole time-of-flight mass spectrometer (SCIEX Triple TOF 5600) coupled to hydrophilic interaction chromatography via electrospray ionization in Shanghai Applied Protein Technology Co., Ltd.

When liquid chromatographic separation was achieved on the HILIC column. The MS conditions were as follows: ion spray voltage was +5.5/-5.5 kV (positive/negative); the turbo ion spray temperature was 600 °C; voltage nebulizer gas, 60 psi; heater gas, 60 psi; curtain gas, 30 psi. The full-scan m/z ranging from 25 to 1000 with a 0.05 s/spectra ion accumulation time was employed using the information-dependent acquisition mode.

When liquid chromatographic separation was achieved on HSS T3 column. The MS conditions were as follows: ion spray voltage was +5.0/-5.0 kV (positive/negative); the turbo ion spray temperature was 650 °C; voltage nebulizer gas, 40 psi; heater gas, 80 psi; curtain gas, 30 psi. The full-scan m/z ranging from 25 to 1000 with a 0.05 s/spectra ion accumulation time was employed using the information-dependent acquisition mode.

## Supplementary Method 4. Data processing

Compound identification of metabolites by MS/MS spectra with an in-house database established with available authentic standards. The matching database used is the local self-built standard database (Shanghai Applied Protein Technology). The number is 30000+, and 6000+ metabolites are self-built by us with standards (including 4000+ for human and animal). Plants 2000+), others from 4 public databases: Mass Bank, Metlin, HMDB, and MoNA corresponding secondary standard spectrograms. In this part of the public database, only this part of the spectrum collected on the high-resolution mass spectrum is retained, the low-resolution mass spectrum and this part of the low-quality spectrum with problems in the quality of the spectrum are filtered out, and then the high-quality spectrum is checked manually to confirm that there is no problem, and these metabolites are integrated into the 30000 + database.

In data analysis, the Variable Importance for the Projection (VIP) value of each variable in the OPLS-DA model was calculated to indicate its contribution to the classification. Significance was determined using an unpaired Student’s t-test. VIP value >1 and p<0.05 was considered statistically significant.

# Supplementary Figures and Tables

## Supplementary Figures

**
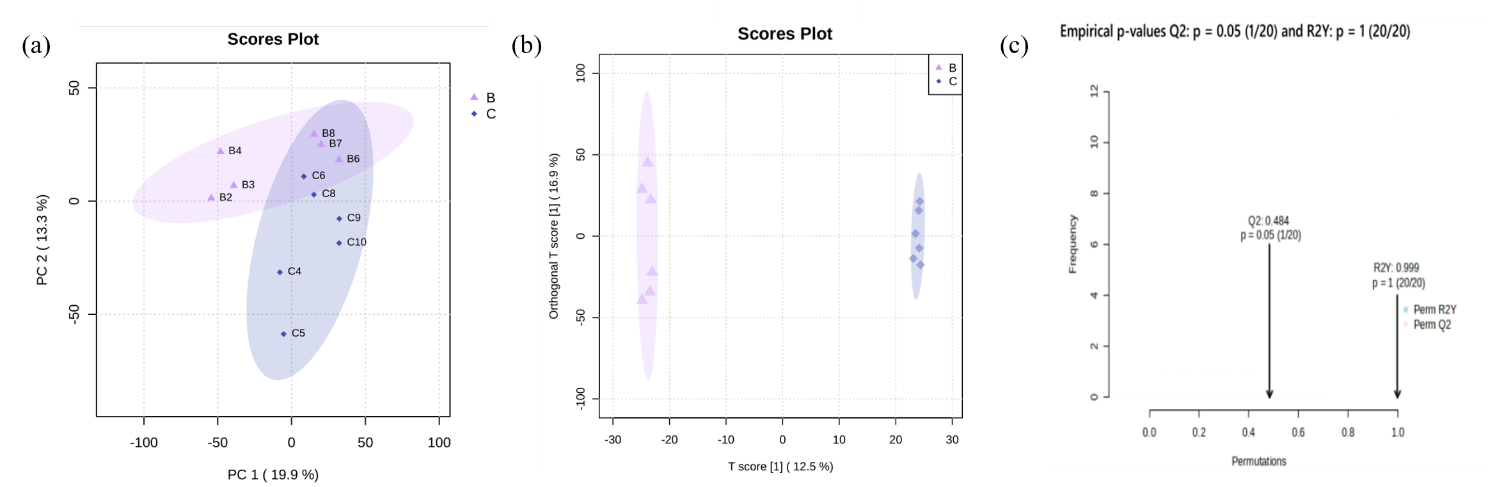
**

**Supplementary Figure S1. PCA values and OPLS-DA scores** (a)-(c) PCA values and OPLS-DA scores between samples C and B indicate the differences between them. Group C and Group B in the HILIC negative and positive ion detection modes.


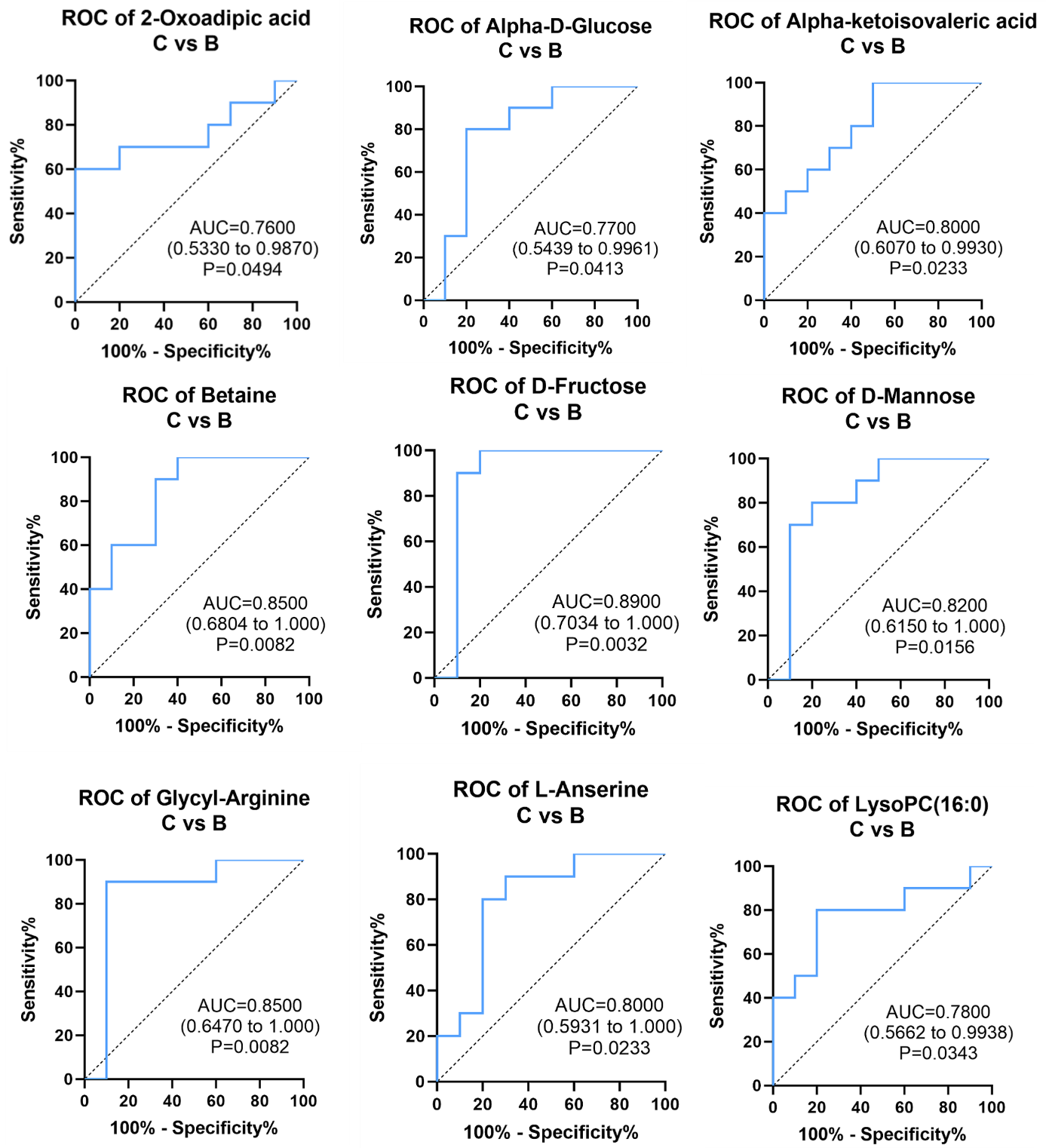


**Supplementary Figure S2. Evaluation of the effectiveness of 30 metabolite indicators for classification and diagnosis in two groups (Group C and Group B).** The graph shows the 9 metabolites with the most significant indicators.

## Supplementary Tables

### Table S1 Diet composition and feed consumption

Composition of experimental diets for three groups of rats

| **Ingredient (%)** | **Standard Diet** |
| --- | --- |
| Dry matter | 90.3 |
| Protein | 23.0 |
| Moisture | 9.7 |
| Fat | 4.7 |
| Ash | 6.1 |
| Fibre | 4.4 |
| Calories (Kcal/g) | 3.4 |

Average weekly feed consumption of three groups of rats (n=15)

| **Feed Consumption (weeks)** | **Control A (g)** | **Group B (g)** | **Group C (g)** |
| --- | --- | --- | --- |
| 1 | 109.9 g | 114.1 g | 100.1 g |
| 2 | 149.8 g | 149.1 g | 137.2 g |
| 3 | 151.9 g | 154.7 g | 144.9 g |
| 4 | 164.5 g | 160.3 g | 160.3 g |
| 5 | 151.2 g | 159.6 g | 153.3 g |
| 6 | 154.1 g | 159.6 g | 145.6 g |
| 7 | 163.8 g | 171.5 g | 177.1 g |
| 8 | 178.5 g | 168.7 g | 162.4 g |
| 9 | 179.7 g | 167.3 g | 172.2 g |
| 10 | 169.4 g | 167.3 g | 162.4 g |
| 11 | 173.6 g | 171.5 g | 167.3 g |
| 12 | 163.8 g | 169.4 g | 175.0 g |
| 13 | 165.2 g | 172.2 g | 165.9 g |

### Table S2 Statistical analysis of 15 indicators in three groups of rats (n=10)

Variables

**Sex Control A Group B Group C**

| Male | 10 | 10 | 10 |
| --- | --- | --- | --- |

**ALT**

| Mean±SD | 54.9±15.7 | 50.1±9.9 | 51.4±9.9 |
| --- | --- | --- | --- |
| Median | 51.5 | 47.3 | 50.3 |
| Range | 40.1-98.5 | 42.2-82.4 | 42.2-82.4 |

**AST**

| Mean±SD | 168.6±19.1 | 198.5±60.7 | 168.7±23.4 |
| --- | --- | --- | --- |
| Median | 163.4 | 178.3 | 164.7 |
| Range | 152.3-197.8 | 155.9-370.3 | 134.2-221.1 |

**ALB**

| Mean±SD | 37.1±1.2 | 36.7±1.8 | 37.5±1.3 |
| --- | --- | --- | --- |
| Median | 37.2 | 35.9 | 37.8 |
| Range | 34.9-39.0 | 34.2-39.5 | 34.7-39.7 |

**ALP**

| Mean±SD | 163.8±48.1 | 157.8±54.3 | 151.0±30.9 |
| --- | --- | --- | --- |
| Median | 149.7 | 131.6 | 143.6 |
| Range | 100.6-254.8 | 104.6-285.7 | 96.8-205.7 |

**D-BIL**

| Mean±SD | 23.4±5.1 | 16.1±2.7 | 16.9±6.5 |
| --- | --- | --- | --- |
| Median | 24.4 | 16.5 | 15.8 |
| Range | 16.8-25.3 | 11.9-20.3 | 9.4-31.6 |

**T-BIL**

| Mean±SD | 49.7±11.3 | 33.4±6.8 | 34.7±10.6 |
| --- | --- | --- | --- |
| Median | 45.7 | 36.2 | 33.4 |
| Range | 40.4-74.3 | 22.1-42.4 | 21.7-52.4 |

**γ-GT**

| Mean±SD | 3.7±0.9 | 3.0±1.2 | 3.4±1.0 |
| --- | --- | --- | --- |
| Median | 4.0 | 3.0 | 3.1 |
| Range | 2.1-4.7 | 0.8-4.9 | 2.1-4.9 |

**UA**

| Mean±SD | 0.1±0.0 | 0.1±0.0 | 0.1±0.0 |
| --- | --- | --- | --- |
| Median | 0.1 | 0.1 | 0.1 |
| Range | 0.1-0.1 | 0.1-0.1 | 0.1-0.1 |

**TBA**

| Mean±SD | 12.5±4.7 | 9.5±9.1 | 9.6±4.5 |
| --- | --- | --- | --- |
| Median | 10.8 | 6.6 | 9.9 |
| Range | 8.4-16.8 | 4.3-36.2 | 3.5-17.1 |

**UREA**

| Mean±SD | 6.5±0.6 | 6.4±0.5 | 6.5±0.7 |
| --- | --- | --- | --- |
| Median | 6.4 | 6.3 | 6.4 |
| Range | 5.7-7.7 | 5.8-7.5 | 5.6-8.0 |

**CREA**

| Mean±SD | 38.5±4.2 | 41.2±3.3 | 36.3±2.6 |
| --- | --- | --- | --- |
| Median | 37.1 | 41.4 | 36.9 |
| Range | 33.3-45.9 | 36.8-46.4 | 29.6-39.0 |

**CK**

| Mean±SD | 307.9±74.7 | 456.2±143.9 | 355.5±78.5 |
| --- | --- | --- | --- |
| Median | 317.4 | 436.55 | 346.3 |
| Range | 143.2-405.2 | 312.2-777.8 | 220.9-498 |

**CK-MB**

| Mean±SD | 17.7±3.9 | 21.3±5.4 | 18.9±4.1 |
| --- | --- | --- | --- |
| Median | 17.4 | 20.2 | 18.7 |
| Range | 13-28.2 | 14.7-27.9 | 10.4-25.0 |

**LDH**

| Mean±SD | 1651.0±270.6 | 1962.6±457.2 | 1580.5±276.8 |
| --- | --- | --- | --- |
| Median | 1633.7 | 1797.5 | 1590.8 |
| Range | 1319.3-2255.6 | 1455.7-2717 | 1012.2-1941.9 |

**LDH1**

| Mean±SD | 13±3 | 14±2 | 12±3 |
| --- | --- | --- | --- |
| Median | 13 | 15 | 12 |
| Range | 10-19 | 10-17 | 3-16 |

### Table S3 The detailed results of significantly differential metabolites in the positive and negative detection mode with the comparison of Control A with Group B (VIP> 1.0 and P <0.05)

| **Name** | **adduct** | **VIP** | **FC** | **P-value** | **m/z** | **rt(s)** |
| --- | --- | --- | --- | --- | --- | --- |
| 1-Stearoyl-sn-glycerol 3-phosphocholine | (M-H+2Na)^+^ | 5.25 | 0.71 | 0.0075 | 568.34 | 314.63 |
| Acetylcarnitine | (M+H)^+^ | 8.98 | 1.37 | 0.0074 | 204.12 | 572.55 |
| Anthranilic acid (Vitamin L1) | (M+H)^+^ | 2.60 | 0.64 | 0.0050 | 138.05 | 543.20 |
| Betaine | (M+H)^+^ | 1.88 | 1.31 | 0.0404 | 118.09 | 565.08 |
| Cytidine | (M+H)^+^ | 1.58 | 1.47 | 0.0008 | 244.09 | 446.94 |
| Cytosine | (M+H)^+^ | 1.25 | 1.34 | 0.0020 | 112.05 | 446.84 |
| L-Glutamine | (M+Na)^+^ | 1.01 | 1.26 | 0.0455 | 169.06 | 721.43 |
| L-Leucine | (M+H)^+^ | 3.15 | 1.19 | 0.0098 | 132.10 | 486.25 |
| L-Methionine | (M+H)^+^ | 1.71 | 1.61 | 0.0365 | 150.06 | 534.34 |
| Thymidine | (M+H)^+^ | 1.28 | 1.22 | 0.0226 | 243.10 | 145.57 |
| 2-Oxoadipic acid | (M-H_2_O-H)^-^ | 12.20 | 1.44 | 0.0434 | 141.02 | 1106.79 |
| 3,4-Dihydroxybenzoate (Protocatechuic acid) | (M-H)^-^ | 1.76 | 0.53 | 0.0010 | 153.02 | 37.28 |
| beta-Nicotinamide D-ribonucleotide | (M+Na-2H)^-^ | 1.30 | 0.65 | 0.0049 | 356.03 | 661.19 |
| D-Quinovose | (M+Na-2H)^-^ | 1.25 | 1.80 | 0.0485 | 185.04 | 394.90 |
| Indoxyl sulfate | (M-H)^-^ | 5.86 | 0.68 | 0.0057 | 212.00 | 40.26 |
| Salicylic acid | (M-H)^-^ | 1.50 | 0.53 | 0.0009 | 137.02 | 52.30 |
| Taurochenodeoxycholate | (M-H)^-^ | 2.41 | 1.53 | 0.0443 | 498.29 | 79.34 |
| 1-Palmitoyl-2-hydroxy-sn-glycero-3-phosphoethanolamine | (M+H)^+^ | 2.37 | 0.79 | 0.0002 | 454.29 | 685.81 |
| D-Threitol | (M+H-2H_2_O)^+^ | 1.06 | 1.14 | 0.0074 | 87.04 | 543.08 |
| gamma-L-Glutamyl-L-glutamic acid | (M+H-H_2_O)^+^ | 1.01 | 1.36 | 0.0082 | 259.09 | 130.10 |
| L-Pyroglutamic acid | (M+H)^+^ | 3.79 | 1.17 | 0.0089 | 130.05 | 130.10 |
| Diethanolamine | (M+H-2H_2_O)^+^ | 1.11 | 0.86 | 0.0179 | 70.06 | 62.37 |
| L-Valine | (M+H)^+^ | 4.03 | 1.12 | 0.0314 | 118.09 | 83.86 |
| 1-Stearoyl-2-hydroxy-sn-glycero-3-phosphoethanolamine | (M+H)^+^ | 1.13 | 0.86 | 0.0321 | 482.32 | 771.55 |
| 1-Myristoyl-sn-glycero-3-phosphocholine | (M+H)^+^ | 3.15 | 0.83 | 0.0337 | 468.31 | 616.53 |
| Thioetheramide-PC | (M+Na)^+^ | 2.65 | 0.58 | 0.0454 | 758.57 | 726.75 |
| LysoPC(16:0) | (M+Na)^+^ | 2.30 | 0.71 | 0.0325 | 518.32 | 326.93 |
| LysoPC(18:1(9Z)) | (M+H)^+^ | 5.56 | 0.83 | 0.0421 | 522.35 | 348.72 |
| LysoPE(16:0/0:0) | (M+H)^+^ | 2.83 | 0.63 | 0.0052 | 454.29 | 344.88 |
| PC(16:0/16:0) | (M+Na)^+^ | 2.60 | 0.75 | 0.0036 | 756.55 | 258.87 |

### Table S4 The detailed results of differential metabolites in the positive and negative detection mode with the comparison of Group B with Control A (VIP> 1.0 and 0.05<P<0.1)

| **Name** | **adduct** | **VIP** | **FC** | **P-value** | **m/z** | **rt(s)** |
| --- | --- | --- | --- | --- | --- | --- |
| Allantoin | (M+H)^+^ | 1.78 | 1.16 | 0.0979 | 159.05 | 333.07 |
| D-Proline | (M+H)^+^ | 2.21 | 0.87 | 0.0734 | 116.07 | 588.34 |
| Indolelactic acid | (M+H-H_2_O)^+^ | 2.41 | 1.18 | 0.0712 | 188.07 | 469.75 |
| L-Phenylalanine | (M+H)^+^ | 2.51 | 1.18 | 0.0856 | 166.09 | 472.37 |
| L-Pyroglutamic acid | (M+H)^+^ | 1.96 | 1.12 | 0.0755 | 130.05 | 702.13 |
| PC(18:1(9Z)/18:1(9Z)) | (M+H-H_2_O)^+^ | 1.21 | 1.25 | 0.0662 | 768.59 | 79.03 |
| 4-Pyridoxic acid | (M-H)^-^ | 1.10 | 1.56 | 0.0962 | 182.05 | 53.87 |
| Arachidonic Acid (peroxide free) | (M-H)^-^ | 11.17 | 1.33 | 0.0643 | 303.23 | 65.44 |
| DL-lactate | (M-H)^-^ | 6.94 | 1.20 | 0.0818 | 89.02 | 378.36 |
| Glycocholic acid | (M-H)^-^ | 1.41 | 0.29 | 0.0896 | 464.30 | 430.44 |
| Hippuric acid | (M-H)^-^ | 1.52 | 0.70 | 0.0699 | 178.05 | 356.59 |
| L-Gulonic gamma-lactone | (M-H)^-^ | 2.50 | 0.64 | 0.0912 | 177.04 | 142.11 |
| L-Carnitine | (M+H)^+^ | 1.61 | 0.89 | 0.0563 | 162.11 | 59.43 |
| Ile-Tyr | M^+^ | 1.87 | 1.38 | 0.0564 | 294.15 | 244.83 |
| L-Arginine | (M+H)^+^ | 1.37 | 0.68 | 0.0575 | 175.12 | 54.79 |
| 1-O-Octadecyl-sn-glyceryl-3-phosphorylcholine | (M+K)^+^ | 1.57 | 0.70 | 0.0578 | 548.36 | 694.70 |
| Isomaltose | (M+H-H_2_O)^+^ | 1.94 | 0.80 | 0.0583 | 325.11 | 55.90 |
| Phenylacetylglycine | (M+H)^+^ | 1.01 | 2.07 | 0.0746 | 194.08 | 373.69 |
| 1-Oleoyl-sn-glycero-3-phosphocholine | (M+H)^+^ | 13.97 | 0.83 | 0.0762 | 522.36 | 708.96 |
| L-Palmitoylcarnitine | (M+H)^+^ | 3.30 | 1.32 | 0.0822 | 400.34 | 710.37 |
| DL-3-Hydroxybutyric acid | (M+H-H_2_O)^+^ | 1.05 | 1.78 | 0.0864 | 87.04 | 177.04 |
| Ile-Glu | (M+H)^+^ | 1.12 | 1.21 | 0.0898 | 261.14 | 310.85 |
| Val-Tyr | M+ | 1.31 | 1.34 | 0.0910 | 280.14 | 114.53 |
| Cholic acid | (M+CH_3_COO)^-^ | 1.66 | 0.20 | 0.0539 | 467.30 | 446.76 |
| D(-)-beta-hydroxybutyric acid | (M-H)^-^ | 1.07 | 2.01 | 0.0730 | 103.04 | 52.58 |
| L-Rhamnose | (M+Na-2H)^-^ | 1.02 | 2.34 | 0.0771 | 185.04 | 52.75 |
| Eicosapentaenoic acid | (M-H)^-^ | 1.44 | 1.46 | 0.0780 | 301.22 | 763.40 |
| L-Isoleucine | (M-H)^-^ | 1.94 | 1.37 | 0.0837 | 130.09 | 105.29 |
| alpha-Linolenic acid | (2M-H)^-^ | 1.00 | 1.86 | 0.0892 | 555.44 | 776.07 |
| L-Tryptophan | (M-H)^-^ | 1.65 | 1.15 | 0.0961 | 203.08 | 285.92 |

### Table S5 The detailed results of significantly differential metabolites in the positive and negative detection mode with the comparison of Group C with Group B (VIP>1.0 and P<0.05)

| **Name** | **adduct** | **VIP** | **FC** | **P-value** | **m/z** | **rt(s)** |
| --- | --- | --- | --- | --- | --- | --- |
| Betaine | (M+H)^+^ | 8.13 | 1.12 | 0.0050 | 118.09 | 507.39 |
| D-Mannose | (M+NH_4_)^+^ | 8.37 | 0.69 | 0.0131 | 198.10 | 580.83 |
| D-Proline | (M+H)^+^ | 2.53 | 1.17 | 0.0336 | 116.07 | 588.34 |
| Glycyl-Arginine | (M+CH_3_CN+Na)^+^ | 1.65 | 0.50 | 0.0211 | 295.15 | 875.41 |
| L-Anserine | (M+H)^+^ | 1.87 | 0.54 | 0.0197 | 241.13 | 785.91 |
| L-Leucine | (M+H)^+^ | 1.45 | 0.72 | 0.0050 | 132.10 | 538.48 |
| LysoPC(16:0) | (M+H)^+^ | 30.62 | 1.29 | 0.0364 | 496.34 | 328.42 |
| PC(18:1(9Z)/18:1(9Z)) | (M+H-H_2_O)^+^ | 2.00 | 0.71 | 0.0074 | 768.59 | 251.78 |
| Phenylalanyl-Tyrosine | M^+^ | 1.24 | 0.68 | 0.0469 | 328.14 | 576.14 |
| Tyramine | (M+H-H_2_O)^+^ | 1.94 | 1.17 | 0.0262 | 120.08 | 471.23 |
| 2-Oxoadipic acid | (M-H_2_O-H)^-^ | 6.27 | 1.57 | 0.0297 | 141.02 | 1054.16 |
| Alpha-D-Glucose | (M-H)^-^ | 11.32 | 0.86 | 0.0255 | 179.06 | 560.36 |
| Alpha-ketoisovaleric acid | (M-H)^-^ | 4.18 | 0.57 | 0.0335 | 115.04 | 92.23 |
| D-Fructose | (M+CH_3_COO)^-^ | 7.39 | 0.69 | 0.0028 | 239.08 | 545.37 |
| Dihydrothymine | (M+CH_3_COO)^-^ | 1.08 | 0.68 | 0.0221 | 187.07 | 639.91 |
| D-Ribose | (M-H)^-^ | 1.91 | 0.75 | 0.0293 | 149.05 | 560.35 |
| Glycocholic acid | (M-H)^-^ | 1.48 | 3.58 | 0.0336 | 464.30 | 430.44 |
| Indoxyl sulfate | (M-H)^-^ | 3.21 | 1.29 | 0.0288 | 212.00 | 40.26 |
| LysoPE(16:0/0:0) | (M-H)^-^ | 1.24 | 1.26 | 0.0169 | 452.28 | 360.67 |
| Uracil | (M-H)^-^ | 1.30 | 1.20 | 0.0315 | 111.02 | 142.93 |
| Ile-Tyr | M^+^ | 3.26 | 0.59 | 0.0030 | 294.15 | 244.83 |
| Val-Tyr | M^+^ | 2.33 | 0.60 | 0.0032 | 280.14 | 114.53 |
| Phe-Tyr | M^+^ | 1.58 | 0.63 | 0.0046 | 328.14 | 294.44 |
| Pro-Asp | (M+CH_3_COO+2H)^+^ | 1.68 | 0.65 | 0.0099 | 291.12 | 58.80 |
| L-Valine | (M+H)^+^ | 3.05 | 0.92 | 0.0497 | 118.09 | 83.86 |

### Table S6 The detailed results of differential metabolites in the positive and negative detection mode with the comparison of Group C with Group B (VIP>1.0 and 0.05<P<0.1)

| **Name** | **adduct** | **VIP** | **FC** | **P-value** | **m/z** | **rt(s)** |
| --- | --- | --- | --- | --- | --- | --- |
| Glycocholic acid | (M+H)^+^ | 1.02 | 2.80 | 0.0561 | 466.31 | 476.50 |
| Indolelactic acid | (M+H-H_2_O)^+^ | 2.30 | 0.90 | 0.0663 | 188.07 | 469.75 |
| Isoleucyl-Tyrosine | M^+^ | 1.01 | 0.70 | 0.0999 | 294.15 | 602.02 |
| L-Gulonic gamma-lactone | (M+NH_4_)^+^ | 2.82 | 0.70 | 0.0796 | 196.08 | 213.84 |
| L-Phenylalanine | (M+H)^+^ | 2.12 | 1.22 | 0.0774 | 166.09 | 472.37 |
| LysoPC(18:0) | (M+H)^+^ | 4.45 | 0.87 | 0.0830 | 524.37 | 347.00 |
| D-Threitol | (M+Na-2H)^-^ | 2.14 | 0.78 | 0.0597 | 143.03 | 560.45 |
| ketoisocaproic acid | (M-H)^-^ | 3.53 | 0.48 | 0.0997 | 129.05 | 129.12 |
| Corticosterone | (M+H)^+^ | 2.95 | 0.80 | 0.0709 | 347.22 | 520.36 |
| Isomaltose | (M+H)^+^ | 1.07 | 0.82 | 0.0817 | 343.12 | 55.89 |
| Hippuric acid | (M-H)^-^ | 1.24 | 0.64 | 0.0968 | 178.05 | 255.68 |

### Table S7 The GM yeast dosage of feeding for SD rats

| Weeks | Average Weights (g) | Dose of GM yeasts  (Wet weight, g) | Volume of yeast in buffer (mL) |
| --- | --- | --- | --- |
| 1 | 250 g | 6 | 2.5 |
| 2 | 320 g | 7.68 | 2.5 |
| 3 | 370 g | 8.8 | 3 |
| 4-6 | 420 g | 10 | 3 |
| 7 | 500 g | 12 | 4 |
| 8-10 | 550 g | 13.2 | 4 |
| 11-13 | 600 g | 14.4 | 4 |
